# Cytoplasmic p21 induced by p65 prevents doxorubicin-induced cell death in pancreatic carcinoma cell line

**DOI:** 10.1186/1423-0127-19-15

**Published:** 2012-02-04

**Authors:** YingQi Zhou, Gang Li, Yuan Ji, Chen Liu, JingPing Zhu, YanJun Lu

**Affiliations:** 1 The Third General Surgery Department, Zhanghai Hospital, Second Military Medical University, 168 Zhanghai Road, Shanghai 200433, China; 2 Department of Pathology, Zhongshan Hospital, Fudan University, Shanghai, China; 3 Laboratory of Cancer Research, Tongji University School of Medicine, 1239 Siping Road, Shanghai 200092, China

**Keywords:** p21, p65, p53, caspase-3, DOX, PANC1

## Abstract

**Background:**

Studies have shown the existence of p21 induction in a p53-dependent and -independent pathway. Our previous study indicates that DOX-induced p65 is able to bind the p21 promoter to activate its transactivation in the cells.

**Methods:**

Over-expression and knock-down experiments were performed in Human Pancreatic Carcinoma (PANC1) cells. Cell cycle and cell death related proteins were assessed by Western Blotting. Cytotoxicity assay was checked by CCK-8 kit. Cell growth was analyzed by flow cytometers.

**Results:**

Here we showed that over-expression of p65 decreased the cytotoxic effect of DOX on PANC1 cells, correlating with increased induction of cytoplasmic p21. We observed that pro-caspase-3 physically associated with cytoplasmic p21, which may be contribution to prevent p21 translocation into the nucleus. Our data also suggested that no clear elevation of nuclear p21 by p65 provides a survival advantage by progression cell cycle after treatment of DOX. Likewise, down-regulation of p65 expression enhanced the cytotoxic effect of DOX, due to a significant decrease of mRNA levels of anti-apoptotic genes, such as the cellular inhibitor of apoptosis-1 (c-IAP1), and the long isoform of B cell leukemia/lymphoma-2 (Bcl-2), leading to efficient induction of caspase-3 cleavage in the cells. More, we present evidence that over-expression of p53 or p53/p65 in the PANC1 cells were more sensitive to DOX treatment, correlated with activation of caspase-3 and clear elevation of nuclear p21 level. Our previous data suggested that expression of p21 increases Gefitinib-induced cell death by blocking the cell cycle at the G1 and G2 phases. The present findings here reinforced this idea by showing p21's ability of potentiality of DOX-induced cell death correlated with its inhibition of cell cycle progression after over-expression of p53 or p53/p65.

**Conclusion:**

Our data suggested p65 could increase p53-mediated cell death in response to DOX in PANC1 cells. Thus, it is worth noting that in p53 null or defective tumors, targeting in down-regulation of p65 may well be useful, leading to the potentiality of chemotherapeutic drugs.

## Background

Pancreatic carcinoma is one of the most leading causes of cancer mortality in the worldwide. Due to the aggressive nature of the disease and the difficulties in diagnosis, the overall 5-year survival rate of pancreatic carcinoma is less than 5% [1,2]. Pancreatic cancer has been reported to be resistant to most of chemotherapeutic drugs. The novel approaches for the treatment of pancreatic cancer are necessary to improve the survival rate.

Nuclear factor-κB (NFκB)/p65 are a transcriptional factor involved in the response to various stimuli and play a central role in inflammatory reactions. P65 generally exists as an inactive dimer sequestered in the cytoplasm by an inhibitor protein termed IκB. Activation of p65 translocate into the nucleus, where it binds to the target genes, and promotes transcription [3].

In response to DNA damage induced by cytotoxic agents, the tumor suppressor p53 accumulates and functions as a sequence-specific DNA-binding protein, which positively regulates expression of several genes, including p21 (waf1/cip1/sdl1) [4]. P21 is an important cellular checkpoint protein for G1 and G2 arrest [5-7].

It is widely accepted that p21 is induced by p53-dependent and p53-independent pathway. We and other group reported that a novel potential NFκB/p65 binding site is at position -2008 of the p21 promoter and the binding of NFκB/p65 protein to this κB site results in transactivation of p21 promoter by p65 [8,9].

Doxorubicin (DOX) is the most widely used chemotherapy agent in treatment of tumor. DOX targets DNA topoisomerase II enzyme activity, which involves sequential DNA binding, cleavage of DNA phosphodiester backbone and subsequently causes DNA breaks. Recent data suggest that DOX-induced cell death is interconnected with the machinery of cell cycle control. Progression through G1 or G2 phase and entry into the S or M phase are tightly regulated by cyclin-dependent kinases (CDKs). Alterations in cell cycle control are a universal feature of cancers. Our previous data suggested that the abnormal progression of cell cycle related with p21 is responsible for DOX-induced cell death [10-13].

Genetic and biochemical studies indicate that DOX-induced cell death is triggered by activation of the members of caspase protease family [14-16]. Activation of caspases during apoptosis converts the inactive, pro-enzyme forms of caspases into the active, processed forms which in turn cleave downstream substrates, leading to biochemical events such as DNA fragmentation [17]. Caspase-3 has been implicated in playing a critical role during apoptosis. Although many molecular pathways are involved in the apoptosis-regulatory mechanism, evidence suggests that the cell cycle and DOX-induced cell death may be involved.

In this study, we showed that the over-expression of p65 remarkably decreased the cytotoxic effect of DOX on Human Pancreatic Carcinoma (PANC1) cells, correlating with the increasing of induction for cytoplasmic p21. In contrast, the reduction of p65 by knock-down of p65 enhanced the cytotoxic effect of DOX treatment due to the increase of activation of caspase-3. We also documented that Induction of p65 enhanced the p53-mediated cell death response to DOX in PANC1 cells.

## Methods

### Cells and transfection

Human Pancreatic Carcinoma (PANC1) cells were cultured in DMEM medium supplemented with 10% FBS (Hyclone, logan, UT). Myc-p21-pcDNA4 and pcDNA4-p53 expression vector were generated as described ref7. ShRNA sequence against p65 (CAA AAA AAG GTC ATG GAC GGT CTA TCT CTT GAA TAG ACC GTC CAT GAC CTTT and TCC CAA AGG TCA TGG ACG GTC TAT TCA AGA GAT AGA CCG TCC ATG ACC TTTT) were clone into pSUPER-EGFP1 constructs (OligoEngine, Seattle, WA). The pSUPER-Scramble plasmid (gat ccc cTT CTC CGA ACG TGT CAC GTt tca aga gaA CGT GAC ACG TTC GGA GAA ttt ttg gaa a) was used as the nonsense control [18]. Cells were transiently transfected with expression constructs using Lipofectamine 2000 reagent (Invitrogen, Carisbad, CA).

### Measurement of cell death

Cells were seeded into 96-well plates and transiently transfected 12 h with expression constructs for p65, p53 or shRNA-p65, followed by DOX treatment (2 μg/ml) for 24 h. Then 10 μl of the CCK-8 solution was added to each well of the plates and then incubated for 2 h in the incubator (37°C and 5% CO_2_). Cytotoxicity assay could be used with the CCK-8 solution according to the procedure of Cell Counting Kit-8 (Dojindo Laboratories, Tokyo, Japan). The absorbency was measured at 450 nm using a micro-plate reader (BioTeK).

### Western Blotting and Immunoprecipitation

The nuclear and cytoplasmic extract from the cells were prepared by the Nuclear Extract Kit (Active Motif, Carlsbad, CA). The protein content of the cell lysate was determined by using the Bradford calorimetric assay method (Bio-Rad, Richmond, CA). The 40 μg aliquot of cytoplasmic or nuclear lysate were resolved by 12% polyacrylamide-sodium lauryl sulfate gel electrophoresis and transferred to a Hybond-C Super membrane (Amersham, Buckinghamshire, UK). The antibodies against p65 (8242, Cell Signaling), pro-caspase-3 (9665, Cell Signaling), active-casepase-3 (9664, Cell Signaling), p53 (9282, Cell Signaling), p21 (2946, Cell Signaling), CDK2 (2546, Cell Signaling), CDK4 (2906, Cell Signaling), cyclinD1 (2922, Cell Signaling), cyclinE (4129, Cell Signaling), Myc (46-0603, Invitrogen) and GAPDH (AG019, Beyotime) were used. Then the blot was incubated with a secondary antibody, IRDye 800 conjugated affinity purified anti-mouse or anti-rabbit IgG (Rockland Immunochemical, Inc., Gilbertsville, PA) and detected with Odyssey Infrared Imaging System (LI-COR Biosciences, Nebraska, USA). For immunoprecipitation, a 500 μg aliquot of cell lysate was incubated for 1 h at 4°C with the anti-p21 anti-body (2946, Cell Signaling). Immune complexes were captured by incubation with 80 μl protein G-plus-agarose (Santa Cruz Biotechnology, Santa Cruz, CA) for 10 h, then washed several times with NP-40 buffer and lysed with loading buffer to do Western Blotting analysis.

### Cell growth analysis

Cells were washed twice with PBS, trypsin zed and re-suspended in PBS containing 0.1% Triton X-100 and RNase (1 mg/ml) (Sigma, St Louis, MO). The cell suspension was incubated at 37°C for 30 min. Propidium iodide (Molecular Probes, Inc. Eugene, OR) was added at a final concentration of 50 μg/ml and the cell suspension was kept at 4°C for 1 hour. The cells were filtered and the cell cycle was analyzed by flow cytometry with the FACScan system (Becton Dickinson, Franklin Lakes, NJ).

### mRNA expression

Total RNA of the cells were extracted by homogenization in 1 mL TRIzol reagent (Invitrogen, Carlsbad CA, USA), followed by chloroform re-extraction and isopropanol precipitation. The RNA was quantified with Eppendorf Biophotometer (Eppendorf, Hamburg, Germany). 0.5 μg of total RNA were reverse transcribed using PrimeScriptTM RT reagent Kit (DRR037A, Takara). Real-time PCR analysis was done using primers: Bcl-2, forward: ACC TGC ACA CCT GGA TCCAG; reverse: CTT GTG GCC CAG ATA GGCAC; cIAP-1, forward: CCT GAG CAG CTT GCA AGTGC; reverse: TGA CGG ATG AAC TCC TGTCC. The average of genes was normalized to the levels of GAPDH: forward: GGT CGT ATT GGG CGC CTG GTC ACC, reverse: CAC ACC CAT GAC GAA CAT GGG GGC.

## Results

### Cytoplasmic p21 induced by p65 is necessary to prevent DOX-induced cell death in PANC1 cells

**Figure 1:**
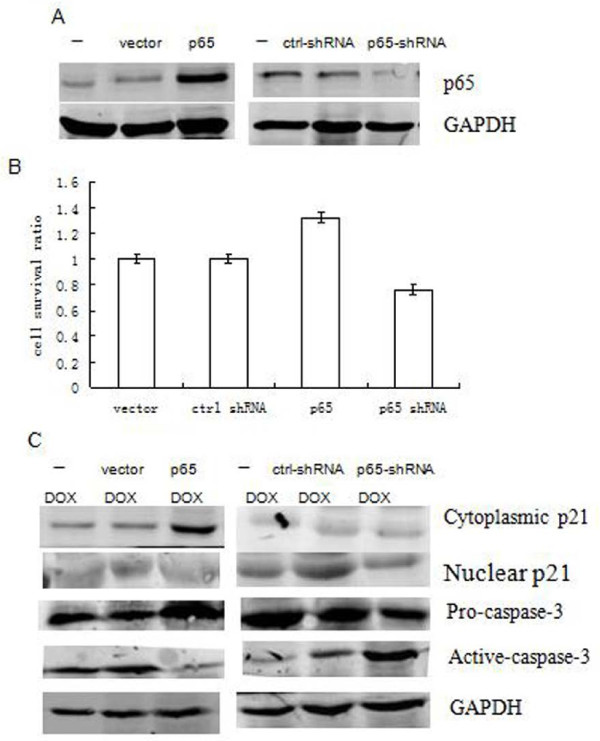
**Cytoplasmic p21 induced by p65 is necessary to prevent the activation of procaspase-3 in PANC1 cell**. Cells were transiently transfected with or without over-expression of p65 and p65-targeted shRNA followed by DOX treatment (2 μg/ml) for 24 h. (A) The level of p65 protein was detected by Western blotting. (B) Cell survival was examined by CCK8 kit. Error bar indicates the standard error of the mean of three independent experiments. (C)The nuclear or cytoplasmic p21 protein (above) and pro-caspase-3 or active caspase-3 (bottom) were detected by Western blotting. GAPDH was used as controls.

NFκB/p65 is a transcription factor that can protect or contribute to apoptosis [19]. To examine the effect of p65 in this study, we transfected with p65 into PANC1 pancreatic cancer cells and detected remarkably increasing of p65 expression in the cells (Figure 1A). Likewise, we used p65-targeted shRNA to silence p65 expression in PANC1 cells. As seen in Figure 1A again, cells transfected with p65-targeted shRNA showed a strong reduction of endogenous p65 expression. Then, the rate of cell survival after treatment with cytotoxic drug DOX (2 μg/ml, 24 h treatment) was shown in Figure 1B. When the PANC1 cells were forced to express p65-targeted shRNA, the percentage of DOX-induced cell death were remarkably increased as compared to control shRNA. In contrast, over-expression of p65 reduced the DOX-induced cell death as compared to vector in PANC1 cells. These data suggested that target in reduction of p65 expression in PANC1 pancreatic cancer cells was useful, leading to the potency of DOX-treatment.

It is the known fact that the expression level of p21 increased in p53-dependent or p53-independent pathway. In previous studies, we showed that p65 increases p21 expression by binding to p21 promoter κB site and p21 (waf1/cip1) is involved in DOX-induced cell death [9]. To investigate the role of p21 in this study, we assessed the cytoplasmic and nuclear levels of p21 in PANC1 cells. As shown in Figure 1C, the cytoplasmic p21 protein expression level in the cell extract was significantly elevated in PANC1 cells after transfection with p65 expression vector followed by DOX treatment for 24 h. However, the nuclear p21 was not changed, as compared to the control vector. In contrast, both cytoplasmic and nuclear levels of p21 were not changed when PANC1 cells were forced to express p65-targeted shRNA (Figure 1C).

**Figure 2:**
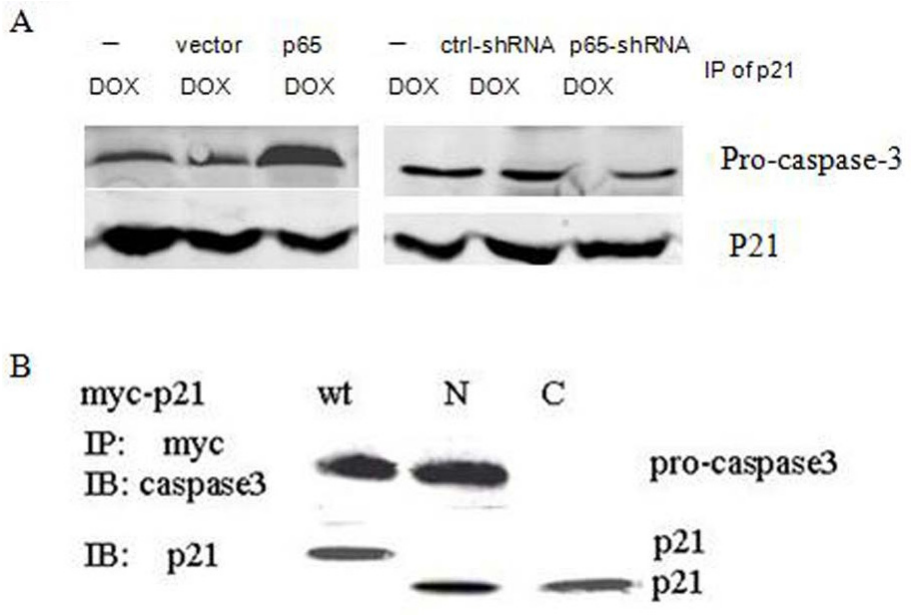
**Pro-caspase-3 physically associated with cytoplasmic p21 induction by p65 in PANC1 cells**. (A) Cells were transiently transfected with or without over-expression of p65 and p65-targeted shRNA followed by DOX treatment (2 μg/ml) for 24 h. Whole cell extract was subjected to immunoprecipitation with anti-p21 antibody and then immunoblotted with anti-pro-caspase-3 or -active-caspase-3 antibody. (B) Cells were transfected with vector expressing wild-type (1-585), N-terminal (1-372) and C-terminal (373-585) region of p21 followed by DOX treatment (2 μg/ml) for 24 h. Immunoprecipitation with an antibody against myc peptide. The pro-caspase-3 and p21 amounts were assessed by Western blotting.

A family of aspartate-specific cysteine proteases (caspases) plays a pivotal role in the execution of programmed cell death. To gain insight into the involvement of caspases in DOX induced cell death in this study, we investigated the effects of p65 expression status on the activation of caspase-3. The protein expression of pro-caspase-3 and active caspase-3 was assessed by Western blotting in PANC1 cells that were treated with DOX at 24 h following p65 over-expression or down-regulation transfection. As shown in Figure 1C again, pro-caspase-3 was significantly elevated in PANC1 cells after transfection with p65 expression vector followed by DOX treatment, as compared to control vector. More, activation of caspase-3 protease cleavage after DOX treatment was suppressed in this condition. In contrast, when the cells were transfected with p65-targeted shRNA, the activation of caspase-3 was increased (Figure 2A). These results demonstrated that activation of caspase-3 was associated with DOX-induced cell death in PANC1 cells (Figure 1B and 1C).

### Pro-caspase-3 physically associated with cytoplasmic p21 induction by p65 in PANC1 cells

The results described above suggest that cytoplasmic p21 induced by p65 prevented cell death induced by DOX treatment in PANC1 cells. We then examined whether there is a biochemical interaction between cytoplasmic p21 and pro-caspase-3 in PANC1 cells. The cytoplasmic p21 proteins were immunoprecipitated from those cells extract using an anti-p21 antibody. The immunoprecipitates were examined by Western blotting with an anti-pro-caspase-3 antibody and an anti-p21 antibody. As shown in Figure 2A, the association between cytoplasmic p21 and pro-caspase-3 was found to remarkably increase when the cells were forced to transiently over-express p65, but not down-regulation of P65. To ask which parts of the p21 participate in interacting with pro-casepase-3. We used with wild-type p21 (1-585), the N-terminal domain of p21 (1-372) and the C-terminal domain of p21 (373-585) expression vectors. These Myc-tagged recombinant p21 proteins were immunoprecipitated from those cells using an anti-Myc peptide antibody. The immunoprecipitates were examined by Western blotting with an anti-pro-caspase-3 antibody and an anti-p21 antibody. Wild-type p21 and the N-terminal domain were found to interact with pr-caspase-3 while the C-terminal domain failed to associate with caspase-3 (Figure 2B). Thus, our results indicate that existing inactivated pro-caspase-3 may prevent p21 translocation from cytoplasm to nucleus by physically associating with cytoplasmic p21 induced by over-expression of p65.

**Figure 3:**
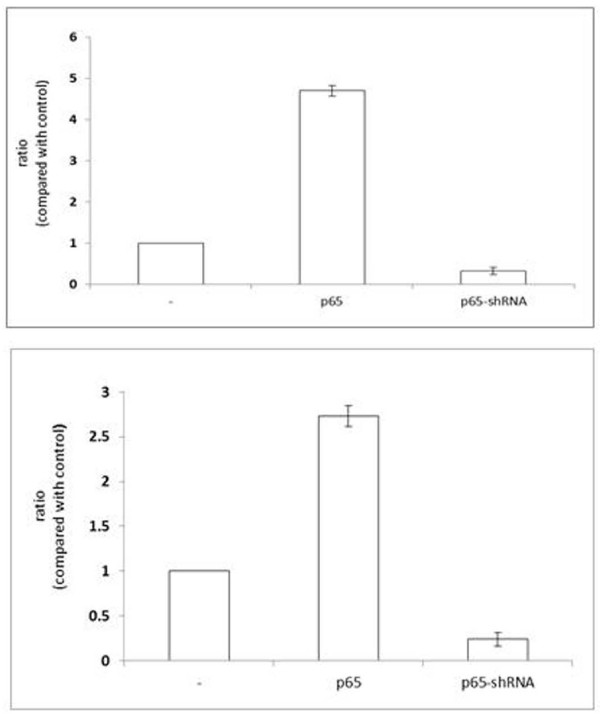
**Down-regulation of p65 reduced expression of the anti-apoptotic genes**. Cells were transiently transfected with or without over-expression of p65 and p65-targeted shRNA. Real-time PCR was performed to assess c-IAP1 (above) and BCL-2 (bottom) mRNA in the cells with GAPDH as control. Error bar indicates the standard error of the mean of three independent experiments.

Next, we were interested in confirming that the effects of the down-regulation of P65 could be seen with p65-regulated target genes. For these experiments, we analyzed cIAP-1 and Bcl-2, which are anti-apoptotic genes regulated by p65. The real-time PCR analysis of RNA extracted from PANC1 cells when the cells were forced to transiently over-express p65 or p65-targeted shRNA. As seen in Figure 3, cIAP-1 and Bcl-2 mRNA levels remarkably increased in PANC1 cells with the over-express p65. In contrast to these observations, down-regulation of P65 resulted in decrease in cIAP-1 and Bcl-2 mRNA levels as compared with its parental PANC1 cells (Figure 3). It led us to suggest that PANC1 cells with down-regulation of P65 were more sensitive to DOX treatment may partly due to decreasing expression of these anti-apoptotic genes.

**Figure 4:**
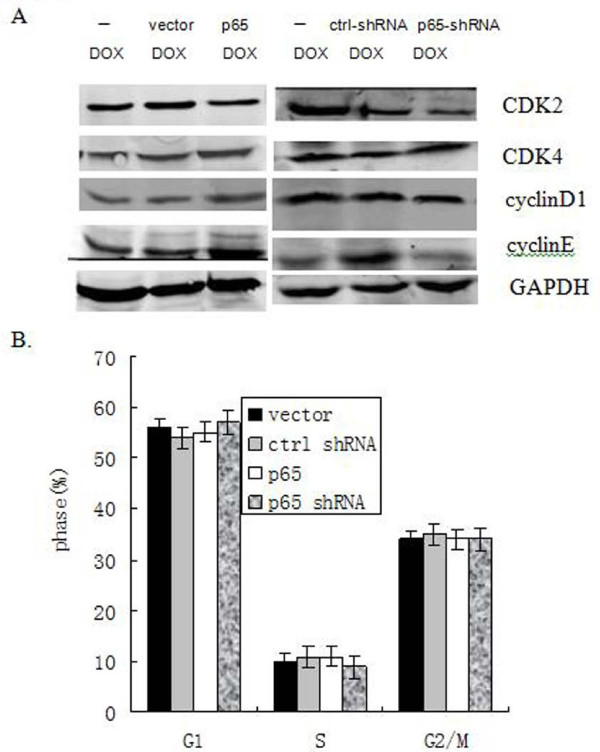
**Change of P65 status was not related cell cycle pattern in PANC1 cells. Cells were transiently transfected with or without over-expression of p65 and p65-targeted shRNA followed by DOX treatment (2 μg/ml) for 24 h**. (A) The level of CDK2, CDK4, and cyclinD1 or cyclinE protein were detected by Western blotting. GAPDH was used as controls. (B) Cell cycle pattern. Error bar indicates the standard error of the mean of three independent experiments.

We further sought to test some other key cell cycle regulators which also showed in Figure 1C. Thus, we observed no remarkably change in the levels of cdk2, cdk4, cyclinE and cyclinD1 by DOX in PANC1 cells with reduction or over-expression of p65 (Figure 4A). These data was consistent with results of cell cycle analysis showed that no change in the cell cycle pattern of these cells after regulating expression of p65 (Figure 4B). Our data suggested that no clear elevation of nuclear p21 by p65 provides a survival advantage by progression cell cycle after treatment of DOX.

### Induction of p65 enhanced the p53-mediated cell death response to DOX in PANC1 cells

**Figure 5:**
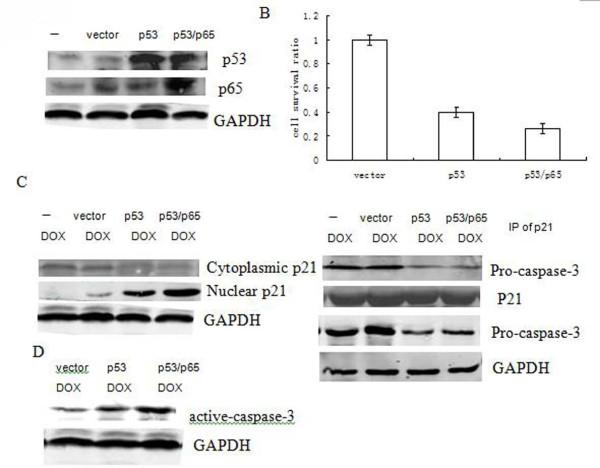
**Induction of p65 enhanced the p53-mediated cell death response to DOX in PANC1 cells**. Cells were transiently transfected with or without over-expression of p53 alone or p53 and p65 together followed by DOX treatment (2 μg/ml) for 24 h. (A) The level of p53 or p65 protein was detected by Western blotting. (B) Cell survival was examined by CCK8 kit. Error bar indicates the standard error of the mean of three independent experiments. (C)The nuclear or cytoplasmic p21 protein (above) or pro-caspase-3 (bottom) were detected by Western blotting. GAPDH was used as controls. Whole cell extract was subjected to immunoprecipitation with anti-p21 antibody and then immunoblotted with anti-pro-caspase-3 antibody (middle). (D) Active-caspase-3 and active-caspase-8 protein were detected by Western blotting. GAPDH was used as controls.

P53 tumor suppressor is known to play an important role in mediating DNA damage agent's induced-cell death through a variety of mechanisms [20-22]. To confirm this point, we over-expressed p53 into PANC1 pancreatic cancer cells which are endogenous p53-negative (Figure 5A). Next, we used DOX to treat PANC1 (p53++) cells and its parental cells for 24 h. As shown in Figure 4B, DOX-induced cell death was remarkably higher in PANC1 (p53++) cells than in its parent cells. This result suggests a requirement of p53 for DOX-induced cell death PANC1 pancreatic cancer cells.

Then we want to check p65 effects on the cells of over-expression of p53, and co-transfected with p53 and p65 into the cells. As shown in Figure 5A again, a clear elevation level of p53 or p65 was seen as compared to the control vector. Furthermore, cell survival rate was decreased after forced over-expression p53 and p65, as compared to the cells transfected with p53 alone (Figure 5B). These data led us to suggest that induction of p65 specifically enhanced the p53-mediated cell death response to DOX.

To investigate the role of p21 in this case, we assessed cytoplasmic and nuclear levels of p21 in these cells. As shown in Figure 5C, the nuclear p21 protein expression level in the cell extract was significantly elevated in PANC1 cells after transfection with p53 or p53/p65 over-expression vector followed by DOX treatment for 24 h. Moreover, the amount of cytoplasmic p21 likely corresponded to its pattern in nucleus (Figure 5C).

Next, we investigated the effects of the activation of caspase-3 on reduction cytoplasmic p21. The cytoplasmic p21 proteins were immunoprecipitated from those cells extract using an anti-p21 antibody. The immunoprecipitates were examined by Western blotting with an anti-pro-caspase-3 antibody and an anti-p21 antibody. As shown in Figure 5C again, the association between cytoplasmic p21 and pro-caspase-3 was found to remarkably reduce when the cells were forced to transiently over-express p53 or p53/p65. These results indicate that over-expression of p53 mediated caspase-3 activation, which may prevent pro-caspase-3 to physically associate with cytoplasmic p21 and then promoted it translocation from the cytoplasm to nucleus.

More, the protein expression of active caspase-3 was assessed by Western blotting assay. As shown in Figure 5D, active-caspase-3 was significantly elevated in PANC1 cells after transfection with p53 or p53/p65 expression vector followed by DOX treatment, as compared to control vector.

**Figure 6:**
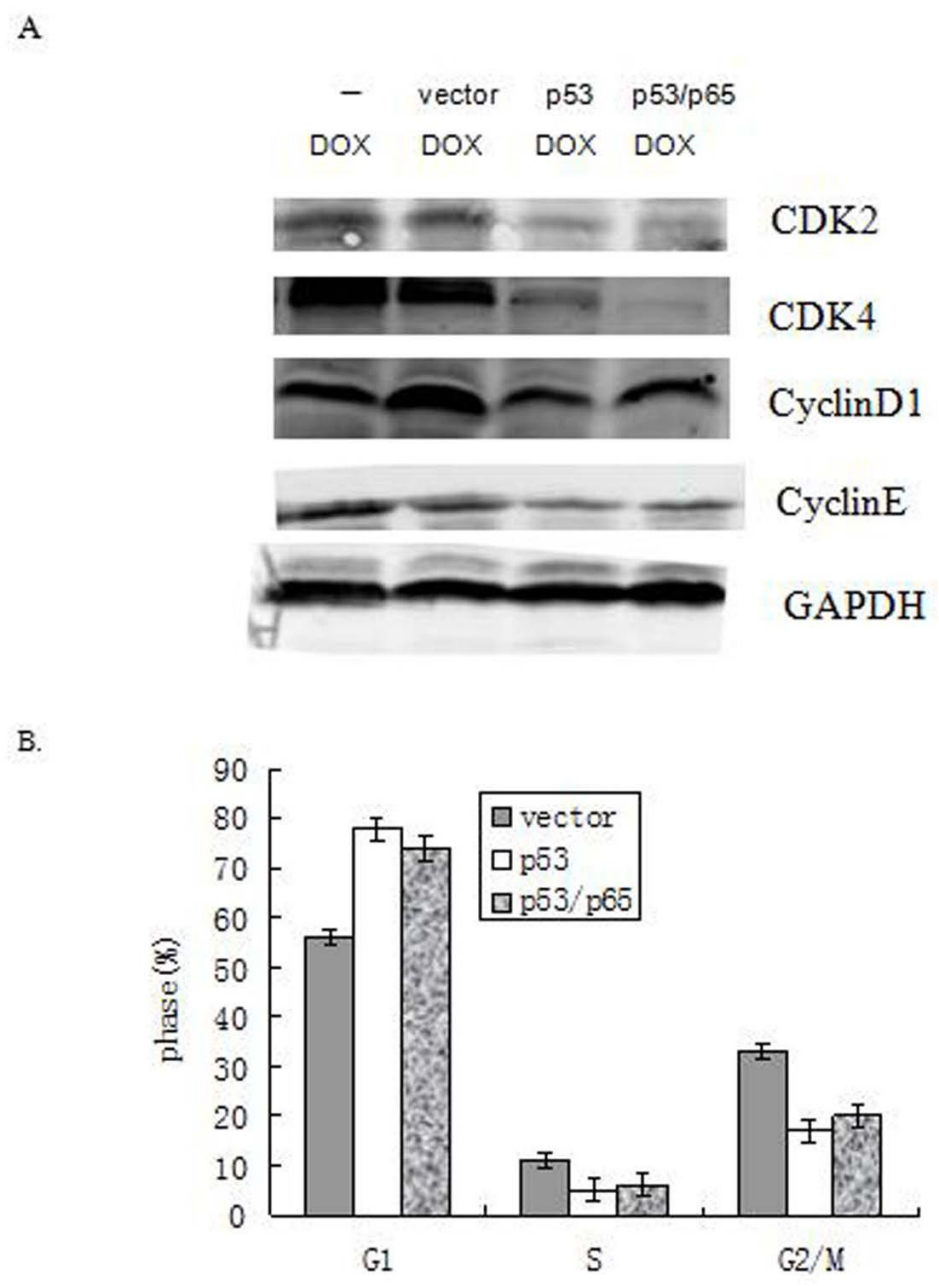
**P53 promoted DOX-induced cell death was partly due to its ability to stop cell cycle progression**. Cells were transiently transfected with or without over-expression of p53 alone or p53 and p65 together followed by DOX treatment (2 μg/ml) for 24 h. (A) The level of CDK2, CDK4, cyclinD1 or cyclinE protein were detected by Western blotting. GAPDH was used as controls. (B) Cell cycle pattern. Error bar indicates the standard error of the mean of three independent experiments.

Next, we sought to test some other key cell cycle regulators which also showed in Figure 6A. Thus, we observed remarkably reduction in the levels of cdk2, cdk4, cyclinE and cyclinD1 by DOX after transfection with p53 or p53/p65 over-expression vector. To examine the change in the cell cycle pattern of these cells, the flow cytometry was carried out (Figure 6B). As expected, in PANC1 (p53++) and (p53++/p65++) cells fractions of G1-phase cells were increased from 54 and 49 to 79% and 76%, indicating the cell cycle is arrested at G1-phase due to increasing of nuclear p21 expression. These data suggest data that nuclear p21 induction by p53 promotes cell death may also due to its ability to stop cell cycle progression, which at least partly contributes to DOX-induced cell death in p53-dependent way.

## Discussion

In this study, we showed that the over-expression of p65 remarkably decreased the cytotoxic effect of DOX on PANC1 cells. That correlated with the increasing of induction of cytoplasmic p21. We observed that pro-caspase-3 physically associated with cytoplasmic p21, which may prevent p21 translocation from cytoplasm. In contrast, down-regulation of p65 enhanced the cytotoxic effect to DOX treatment, due to activation of cleavage of caaspase-3 in the cells. More, we present evidence that over-expression of p53 or p53/p65 in the PANC1 cells were more sensitive to DOX treatment, correlated with activation of caspase-3 and clear elevation of nuclear p21 level. Our data suggested p65 could increase p53-mediated cell death in response to DOX in PANC1 cells.

P65 is involved in the regulation of cell death in several systems [19]. In this study, we found that over-expression of p65 remarkably decreased the cytotoxic effect of DOX on PANC1 cells. In contrast, down-regulation of p65 increased DOX-induced cell death in the cells (Figure 1A and 1B). These observations suggest that a regulatory mechanism for p65 must exist to integrate and coordinate its critical cellular decision-making event. Studies have shown the existence of p21 induction in a p53-independent pathway. Our previous study indicates that DOX-induced p65 is able to bind the p21 promoter to activate its transactivation in the cells. Here we observed p21 induction in p65-dependent mechanism in response to DOX (Figure 1C). Thus, we suggested that induction of p21 by p65 is at least partly responsibility to decrease of DOX-induced cell death in the cells.

Genetic and biochemical studies indicate that DOX-induced cell death is triggered by activation of the members of caspase protease family [14,16-18,22]. Caspase-3 plays a pivotal role in the execution of DOX-induced cell death. We and other groups have reported that p65 prevents drugs-induced cytotoxic action by blocking caspases protease activation [8,9]. These results are consistent with our finding that down-regulation of p65 expression was preceded by > 3-fold reduction of c-IAP1 and Bcl-2 mRNA expression in PANC1 cells (Figure 3), suggesting that the decreasing expression of some anti-apoptotic genes contributed to increasing sensitive to DOX-induced cell death. Here, our results indicated that existing inactivated pro-caspase-3 by p65 may prevent p21 translocation from cytoplasm into nucleus by physically associating with cytoplasmic p21 (Figure 2A and 2B). These data are consistent with those of recent studies showing that pro-caspase-3/p21 complex formation could resist cell death in human tumor cells [15-17]. More, our data suggested that no clear elevation of nuclear p21 by over-expression p65 provides a survival advantage by progression cell cycle after treatment of DOX (Figure 4A and 4B).

PANC1 pancreatic cancer cells are endogenous p53-negative. Thus, our data suggest that induction of p65 in p53 null tumors provides a survival advantage by physically associating with procaspase-3 and preventing p21 translocation from cytoplasm to nucleus in PANC1 cells treated with DOX. On the other hand, it is worth noting that in p53 null or defective tumors, targeting in down-regulation of p65 was found to augment an effective therapeutic response to DOX.

In this study, we showed that pancreatic tumor cells that have over-expression of p53 were more sensitive to DOX-induced cell death (Figure 5A and 5B), correlated with activation of caspase-3 whose induction by DOX in the p53-dependent pathway (Figure 5C). The mechanism through which p53 promotes cell death is also dependent on the Apaf-1/caspase-9 pathway and involves cytochrome C releasing from mitochondria [20]. Several potential downstream mediators such as, CD95, Killer/DR5, PERP, p53AIP1 and Bax are involved in p53-dependent cell death [21,22]. Taken together, we propose that p53 is required for DOX-induced cell death in PANC1 pancreatic cancer cells.

Here, we observed that in response to DOX, p21 level was induced by over-expression of p53 or p53 and p65. Moreover, p53 induced caspase-3 to cleavage to its active form after treated with DOX even occurred to co-transfection with p65. Much less physically associating with procaspase-3 promoted p21 translocation from cytoplasm to nucleus, as evidenced by a clear elevation of nuclear p21 level induced by p53 or p53 and p65 (Figure 5D). However, the biochemical basis of the p21 translocation from cytoplasm to nucleus related with caspase-3 should be clarified in the further studies.

DOX induces both cell cycle arrest and apoptosis, although the detailed mechanism whereby DOX commits cells to apoptotic program is unknown. It has been postulated that the imbalance of the cell cycle signals or failure to arrest the cell cycle may trigger the cell death program. Our previous data suggested that expression of p21 increases Gefitinib-induced cell death by inactivation of cyclin-dependent kinase (CDK) activity, which in turn blocks the cell cycle at the G1 and G2 phases [12]. The present findings here reinforced this idea by showing p21's ability of potentiality of DOX-induced cell death correlated with its inhibition of cell cycle progression after over-expression of p53 or p53/p65 (Figure 6A and 6B).

P53 plays an important role in the tumor therapy. However, it is reported that more than 60% human tumors have lost their wild-type p53 function. Increasing evidence suggests that deficiency of p53 in tumors constitutes resistance to chemotherapy and anti-angiogenic treatment. Hence, efforts have been made to improve the efficacy of anti-tumor therapy on the tumors deficient in p53 expression. Here we suggested that in tumor cells that are p53-null or defeated, down-regulation of p65 may well be useful, leading to the potentiality of chemotherapeutic drugs.

However, in tumors that retain wild-type p53, such targeting may be counterproductive, because p65 potentiality of p53-mediated cell death in this study. The tumor suppressor p53 inhibits cell growth through activation of cell-cycle arrest and apoptosis. Our previous data suggested that p65-induced anti-apoptotic gene expressions are much higher in p53-/- cells than in p53+/+ cells. We and other groups found that the transcriptional activities of p53 and p65 are governed by their relative levels of expression: p65 inhibits p53-dependent transactivation, while p53 expression can also suppress p65 transcriptional activity [8,9]. P53 could limit p65-mediated transactivation and this mutual repression mechanism is due to their limiting common co-activators (p300 and CBP) of transcription [23-26]. Thus, we could speculate that p65 loss the ability to promote to induce anti-apoptotic gene expressions, then turn into pro-apoptotic process in p53+/+ cells, which contributes to p65 augmenting an effective therapeutic response to DOX in p53-mediated way.

## Conclusion

In the present study, we observed that the p21 expression level was a key player in the cell cycle and DOX-induced cell death process. The clear elevation of nuclear p21 expression by p53 or p53 and p65 may provide a good rationale for promoting to DOX treatment in Human Pancreatic Carcinoma. Thus, we propose that it may be important in the design of therapeutic protocols that involve targeting of p21 to mediate pancreatic tumor's sensitivity to the drugs.

## Competing interests disclosure

The authors declare that they have no competing interests.

## Authors' contributions

YQZ carried out the cell culture and transfection experiments and drafted the manuscript. GL carried out cell growth and cell death experiments, and contributed to the experiments plans. YJ participated in constructing all those expression plasmids. CL carried out the Western blotting and Immunoprecipitation assay. JPZ carried out Real-time PCR experiment and revised part of manuscript. YJL designed of the study and wrote the manuscript. All authors read and approved the final manuscript.
